# Application of hydrogels in cancer immunotherapy: a bibliometric analysis

**DOI:** 10.3389/fimmu.2024.1433050

**Published:** 2024-08-13

**Authors:** Xiang Liu, Qiang Zhou, Yue Yang, Erhua Chen

**Affiliations:** ^1^ College of Traditional Chinese Medicine, Jiangsu College of Nursing, Huaian, Jiangsu, China; ^2^ Department of Clinical Pharmacy, Jinling Hospital, Medical school of Nanjing University, Nanjing, China

**Keywords:** hydrogels, cancer immunotherapy, bibliometrics, Citespace, cancer

## Abstract

**Background:**

Cancer immunotherapy has made significant progress in recent years, with numerous studies worldwide. Immunotherapy has had a transformative impact on oncology and autoimmune diseases. In the biomedical arena, hydrogels with good properties are widely used in cancer immunotherapy. Our study used bibliometrics to analyze the changing trends in using hydrogels for cancer immunotherapy.

**Methods:**

From 2013 to 2023, a systematic search was conducted in the Web of Science Core Collection database to identify reviews and articles discussing the applications of hydrogels in cancer immunotherapy. The software CiteSpace was used to visually perform the bibliometric analysis in terms of research trends, countries, institutions, authors, journals, and keywords. Individual authors’ productivity was assessed with the Lotka’s law. The most relevant publication sources were identified by Bradford’s law.

**Results:**

A total of 422 English-language publications related to hydrogels in cancer immunotherapy were collected. The number of annual publications increased rapidly after 2021 and remained constant for the past two years. China published the most articles in this field. The institution with the maximum number of published articles was the Chinese Academy of Sciences in China. Chen. Q was the most prolific author, and Liu. Z was the second most published author. In terms of journal contributions, the journal “Biomaterials” had the highest number of publications (n = 30). Biomaterials, Advanced Functional Materials and Journal of Controlled Release were the most influential journals. Keyword analysis revealed that cancer immunotherapy, drug delivery, immunogenic cell death, tumor microenvironment, injectable hydrogels, and immune checkpoint blockade were the primary research hotspots. In recent 3 years, adoptive T-cell therapy, black phosphorus, cell capture, adaptive cell therapy, tumor microenvironment, photodynamic therapy, and sustained release were the research hotspots in this field. Our study summarizes the objective of hydrogels in cancer immunotherapy in recent years, providing a reference for potential researchers in related field.

**Conclusion:**

This bibliometric analysis shows the progress and trend of research on hydrogels in cancer immunotherapy. This study provides a significant avenue for future investigation into current concerns and trends in research within this field.

## 1 Introduction

Cancer is a highly complex disease characterized not only by the proliferation of malignant cells but also by altered immune responses (1). Suppression and reprogramming of the immune system play a key role in cancer development and progression. Immunotherapy aims to reactivate anticancer immune cells and overcome immune evasion mechanisms. Evidence suggests that the combination of multiple immunotherapeutic approaches may improve therapeutic efficacy (2–4). For effective immunotherapy, the cancer immune microenvironment must be considered. The field of developmental origins of cancer immunotherapy dates back to the 1890s when researchers observed that fatal bacterial infections could have an effective, durable antitumor response in patients with partially resected tumors (5, 6). Immunotherapy is a revolutionary paradigm in cancer treatment, demonstrating the potential to obstruct tumor metastasis and recurrence (7, 8). However, immunotherapy often encounters challenges, including severe immune-related side effects, particularly in solid tumors. Hydrogels are vehicles that can be used as local and sustained drug delivery to cancers, providing a promising platform for encapsulating and releasing small molecule drugs, biomolecules, and cells in a controlled manner. Immunomodulatory hydrogels have the unique ability to enhance immune activation and mitigate systemic toxicity by encapsulating multiple components and localized drug delivery (9). Hydrogels are normally produced by mixing liquid monomers, cross-linkers, and initiators together (10). In terms of raw material, hydrogels can be categorized into natural and synthetic types (11). Agar and gelatin are natural hydrogels, usually derived from natural polymers such as plant polysaccharides and proteins, and sodium polyacrylate gel is a synthetic hydrogel, which is mainly prepared by polymerization (12). The use of hydrogels in regenerative medicine and for reconstructive procedures is common (13). Topical hydrogelsadministration can reduce systemic administration-induced extratumoral toxicity (14, 15). With the in-depth study of hydrogels and their continuous improvement in tumor immunotherapy, hydrogels will become potential drug carriers.

In bibliometrics, the number of publications in a discipline combines philology, statistics, and mathematics (16, 17). Since 1969, bibliometrics has been used to describe and analyze the research trends and progress in specific fields (18). Bibliometric analysis has been widely used in medical research (19). CiteSpace is a popular bibliometric visualization program providing statistical visualization of scientific literature worldwide (20). For the present study, we used the CiteSpace software package to visualize, analyze, and map hydrogels in cancer immunotherapy.

Recently, cancer immunotherapy has attracted considerable attention and has emerged as a breakthrough in cancer therapeutics (21). Considerable attention has been given to the hydrogels for cancer immunotherapy. Up to the present, there has no articles reveal the topical issues and collaborations. To the best of our knowledge, although a number of publications on hydrogels in the field of cancer immunotherapy have been published, our study is the first one to use bibliometric methods to address hydrogels in cancer immunotherapy. This study aimed to summarize the recent progress in cancer immunotherapy with hydrogels and their applications in various cancers. Our study aims to provide an overview of the recent studies on hydrogels in cancer immunotherapy since 2013. We have summarized and discussed research hotspots and the latest advances in this field. Our analysis is necessary to help researchers reveal the emerging trends and research directions in the future. Moreover, this study is purposed to provide insights for the utilization of hydrogels in cancer immunotherapy.

## 2 Materials and methods

### 2.1 Data sources and strategies

Data were retrieved from the Science Citation Index Expand web database in the Web of Science Core Collection (WoS). The comprehensiveness, timeliness, and cutting-edge nature of the papers’ sources were considered. Our study used the advanced search modes (Hydrogels*), (Tumor* or Neoplas* or Cancer*) and (Immunotherapy*). From 2013 to 2023, a total of 422 publications were obtained, all of which were research papers in English language and were downloaded under the name “XXX_download.txt” for further bibliometric visualization and analysis.

### 2.2 Data analysis

In the study, CiteSpace 6.2r6 software was used for image visualization. CiteSpace, developed by Prof. Chaomei Chen, is a tool designed for visualizing academic research (22). It visually displays research trends and movements in a specific field, highlighting key topics, scholars, and institutions. By running the CiteSpace software, we used cluster analysis and burst keyword analysis to investigate research trends and hotspots of hydrogels in the field of cancer immunotherapy. The data were analyzed using Bradford’s law and Lotka’s law, using the “bibliometric package” developed in R-language version R 4.2.3 binary.

## 3 Results

### 3.1 Trend and annual count

**Figure 1:**
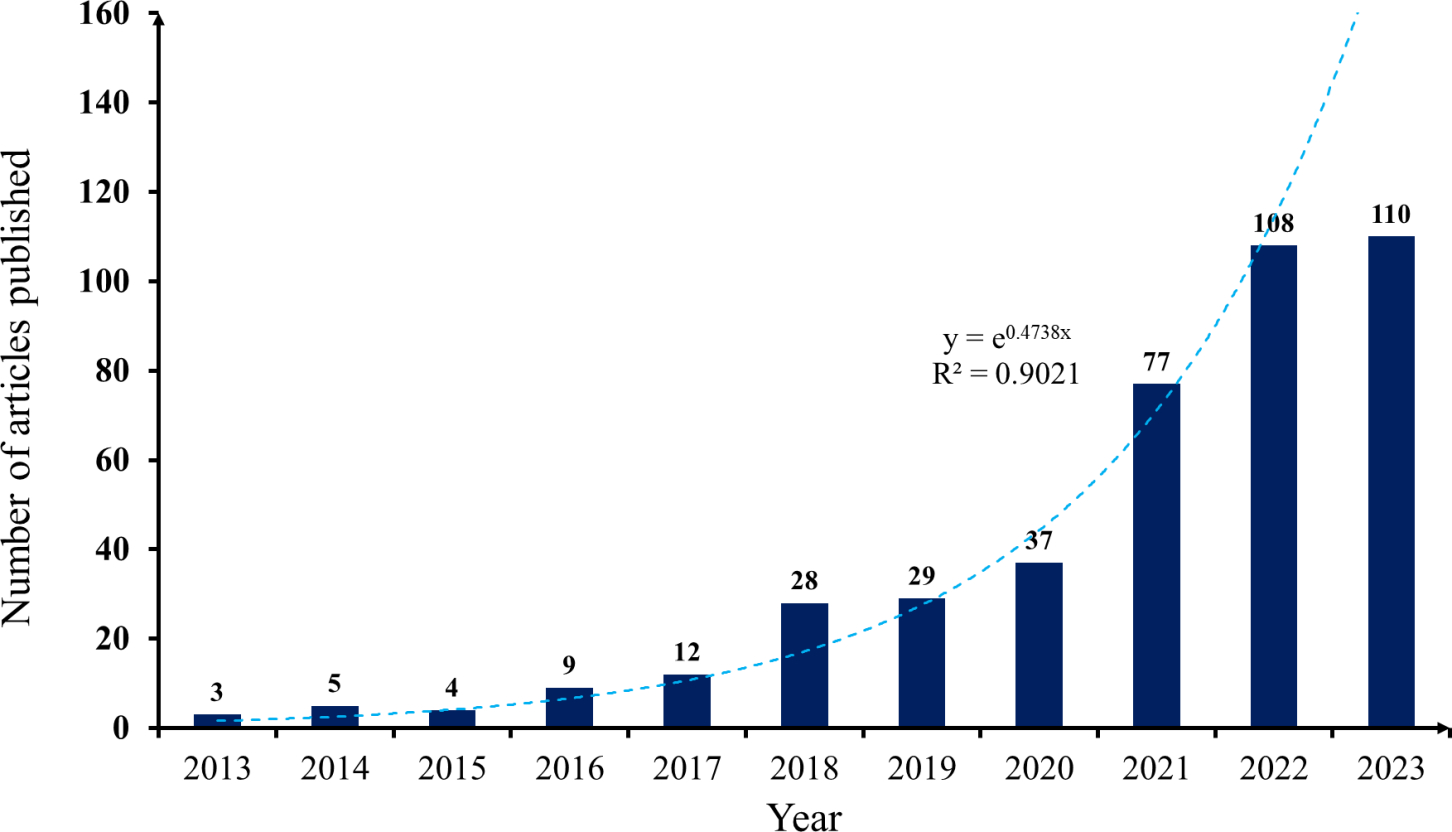
Global publication output and future publication trends of hydrogels in cancer immunotherapy from 2013 to 2023.

The trend of literature publication is an important indicator of the advancement of research in a particular field of study. Therefore, plotting the distribution curve of the amount of literature over time can effectively evaluate the state of research in this area of the discipline and further predict the dynamics and trends of its development. **Figure 1** displays the annual distribution of literature related to hydrogels in cancer immunotherapy research on the WoS for the last 10 years. The average annual number of publications related to hydrogels in cancer immunotherapy research on the WoS for the last 10 years was 38.36. Globally, the use of hydrogels in cancer immunotherapy research has shown an increasing trend. The chronological progression of previous studies showed that the number of publications reached three in 2013, suggesting that the field was in its infancy. The number of scholars focusing on the field has increased over time. During the following decade, the number of studies escalated, and the field matured. The exponential fit index for hydrogels in cancer immunotherapy research is 0.9021, as shown by the trend curve of publications, indicating that there is exponential growth in the current research. Specifically, the average annual number of publications in the early stages of research on the application of hydrogels in cancer immunotherapy during 2013−2016 was approximately 5.25, and the field has received little academic attention. Since 2020, there has been a significant increase in the number of publications in this field. Consequently, with the development of hydrogels in cancer immunotherapy, an increasing number of scholars have begun to pay attention to relevant research. By 2021−2023, the average annual number of publications reached 98.3, which is a growth period of hydrogels application in cancer immunotherapy research. As the application and function of hydrogels in cancer immunotherapy continue to increase, there is a growing academic interest in this area. Consequently, hydrogels will continue to hold considerable promise in the field of cancer immunotherapy research, and their application will continue to expand.

### 3.2 Analysis of article output characteristics

#### 3.2.1 Analysis of countries

**Figure 2:**
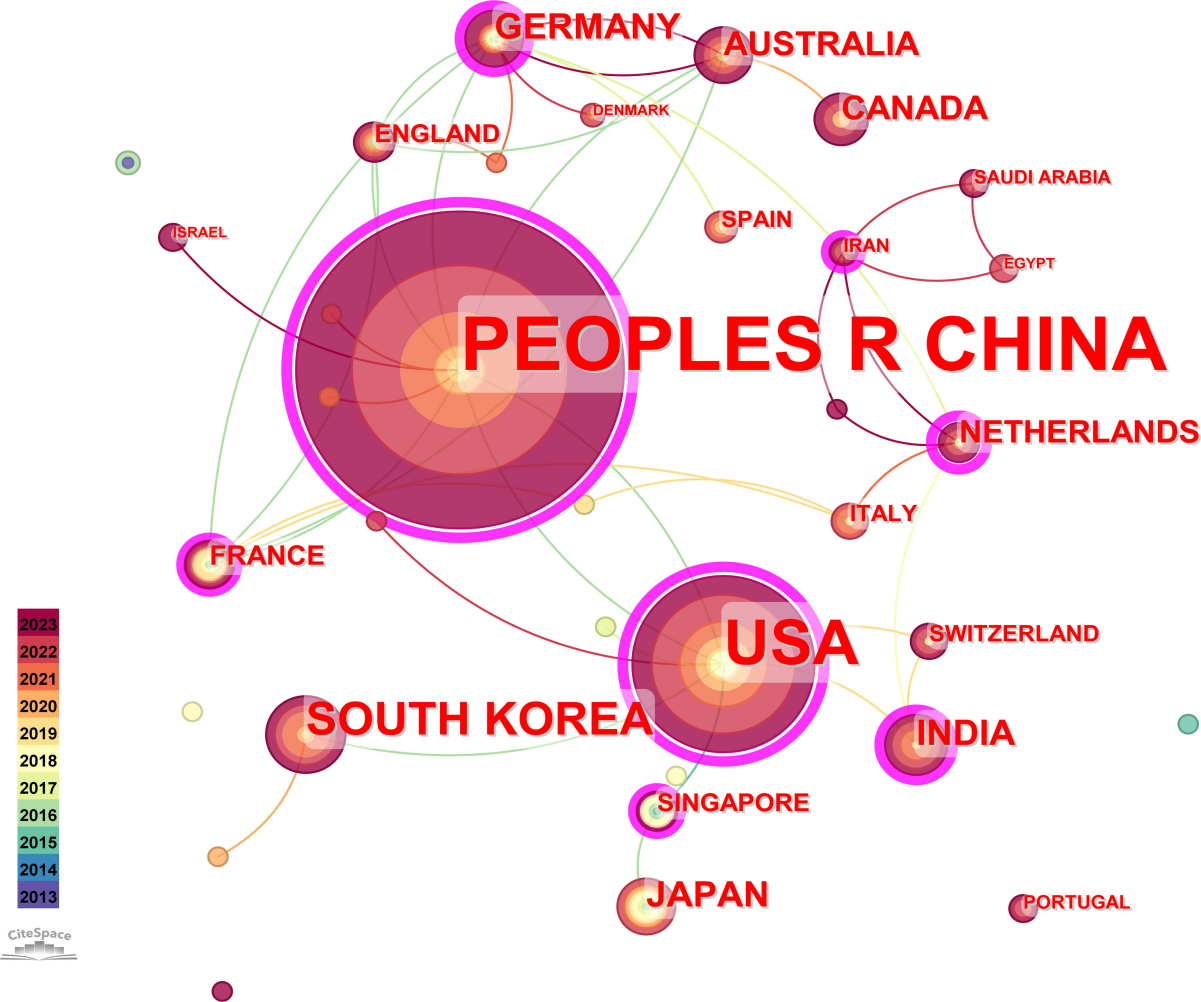
The co-occurrence map of countries.

**Figure 3:**
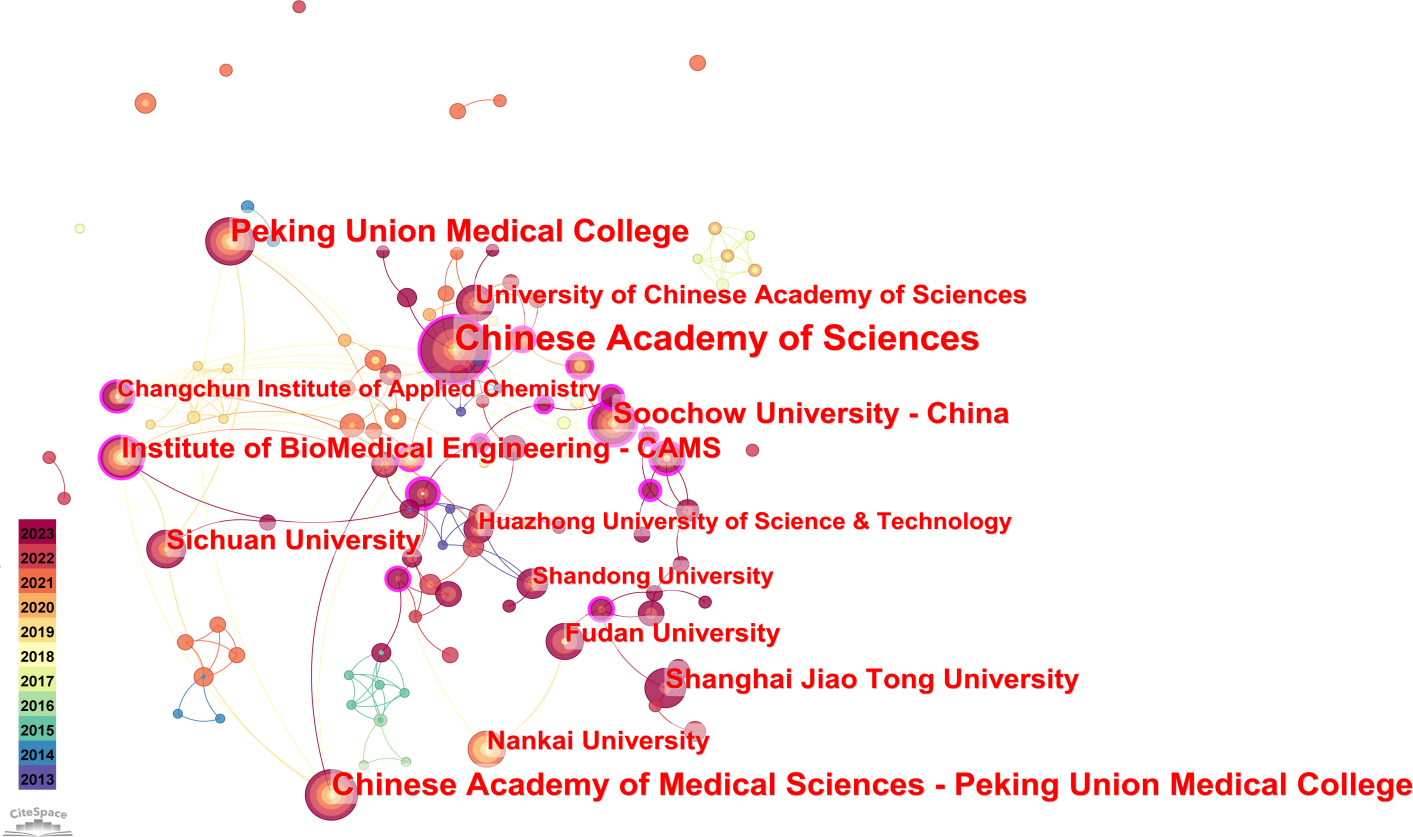
The co-occurrence map of research institutions.

For this study, the country was selected as the analysis object in CiteSpace software; time slicing was set as 2013−2023, years per slice was set as 1, and the g-index = 10. Finally, a national analysis map with 35 network nodes, 44 connections, and a density of 0.0739 was obtained. As displayed in **Figure 2**, the frequency increases with the size of the circle. The different colors reflect the years of study of various countries, and the connections represent the state of cooperation between countries. **Figure 3** illustrates that node circles of China and the United States are the largest, indicating that both countries are high-yielding in the field of hydrogels cancer immunotherapy. Our study also lists the status quo of the top ten countries in terms of frequency (**Table 1**). China has the highest number of publications in this field (n = 269), which is significantly higher than other countries. This indicates that China is highly interested in hydrogels cancer immunotherapy and is expanding its capacity rapidly. The United States exhibited the second-highest frequency at 101, with a significant gap from China, indicating that China is dominant in hydrogels cancer immunotherapy, and other countries are constantly learning and progressing. South Korea ranked third, with a frequency of only 28 publications. From the perspective of centrality, the United States was the highest, reaching 0.74. **Figure 1** demonstrates that the United States has strong connections and cooperates with other countries, which indicates that the United States is at the core of hydrogels cancer immunotherapy. Additionally, similar to other nations, the majority of its research outcomes are derived from collective wisdom. China’s centrality ranks second, reaching 0.62, indicating that China’s position in this field is also important, connecting the cooperative relations of several countries. The centrality of the Netherlands is 0.42, ranking third, indicating that the Netherlands is also growing in this field and is increasingly cooperating with other countries.

**Table 1:** The top 10 countries with the highest frequency.

Rank	Count	Centrality	Year	Country
1	269	0.62	2013	PEOPLES R CHINA
2	101	0.74	2013	USA
3	28	0.1	2013	SOUTH KOREA
4	11	0.39	2016	GERMANY
5	11	0	2015	JAPAN
6	11	0.21	2018	INDIA
7	10	0	2016	CANADA
8	10	0.1	2016	AUSTRALIA
9	8	0.42	2014	NETHERLANDS
10	6	0.12	2016	FRANCE

#### 3.2.2 Analysis of institutions

As part of this study, the institution in CiteSpace was used as the analysis object, and 146 network nodes, 202 connections, and a density of 0.0191 were obtained. As indicated in **Figure 3**, the nodes in the map are relatively close, and the cooperation of each institution can be linked to each institution through connections. This displays frequent institutional cooperation in hydrogels cancer immunotherapy. Most research institutions have fixed partners, and some of them have distinct regional characteristics. Additionally, cross-agency cooperation on the investigation of hydrogel in cancer immunotherapy must be strengthened and broadened.

Our research identified the top ten institutions in hydrogels cancer immunotherapy, and all of them are from China. This indicates that Chinese institutions are paying more attention to research on hydrogels cancer immunotherapy and are high-yield institutional groups in this field (**Table 2**). Particularly, the Chinese Academy of Sciences has the highest number of publications (n = 52). This is because the Chinese Academy of Sciences is the top research institute in the field of science and technology in China and is among the three major research institutes in China. The research in the field of medicine is more extensive, so the number of publications ranks first. Additionally, the number of papers published by the Chinese Academy of Medical Sciences was 32, ranking second. Peking Union Medical College ranked third with 30 publications. By the time of publication, the top ten universities and other institutions had published between 2018 and 2020. This indicates that high-yield institutions were relatively slow to begin investigating hydrogels cancer immunotherapy; however, they have contributed significantly to this field and established the foundation and set the standards. Additionally, it shows that these institutions conducted more extensive research. Soochow University - China has the highest centrality of 0.24, indicating its extensive collaboration with other institutions.

**Table 2:** The top 10 authors with the most publications and greatest centrality.

Rank	Publications	Centrality	Year	Authors
1	14	0.01	2019	Chen, Qian
2	12	0.02	2018	Liu, Zhuang
3	12	0	2018	Wang, Weiwei
4	10	0	2018	Huang, Pingsheng
5	9	0	2018	Song, Huijuan
6	9	0	2018	Kong, Deling
7	9	0	2018	Chen, Xuesi
8	7	0	2018	Zhang, Chuangnian
9	6	0	2018	He, Chaoliang
10	5	0.01	2018	Wang, Chao

#### 3.2.3 Analysis of authors

**Figure 4:**
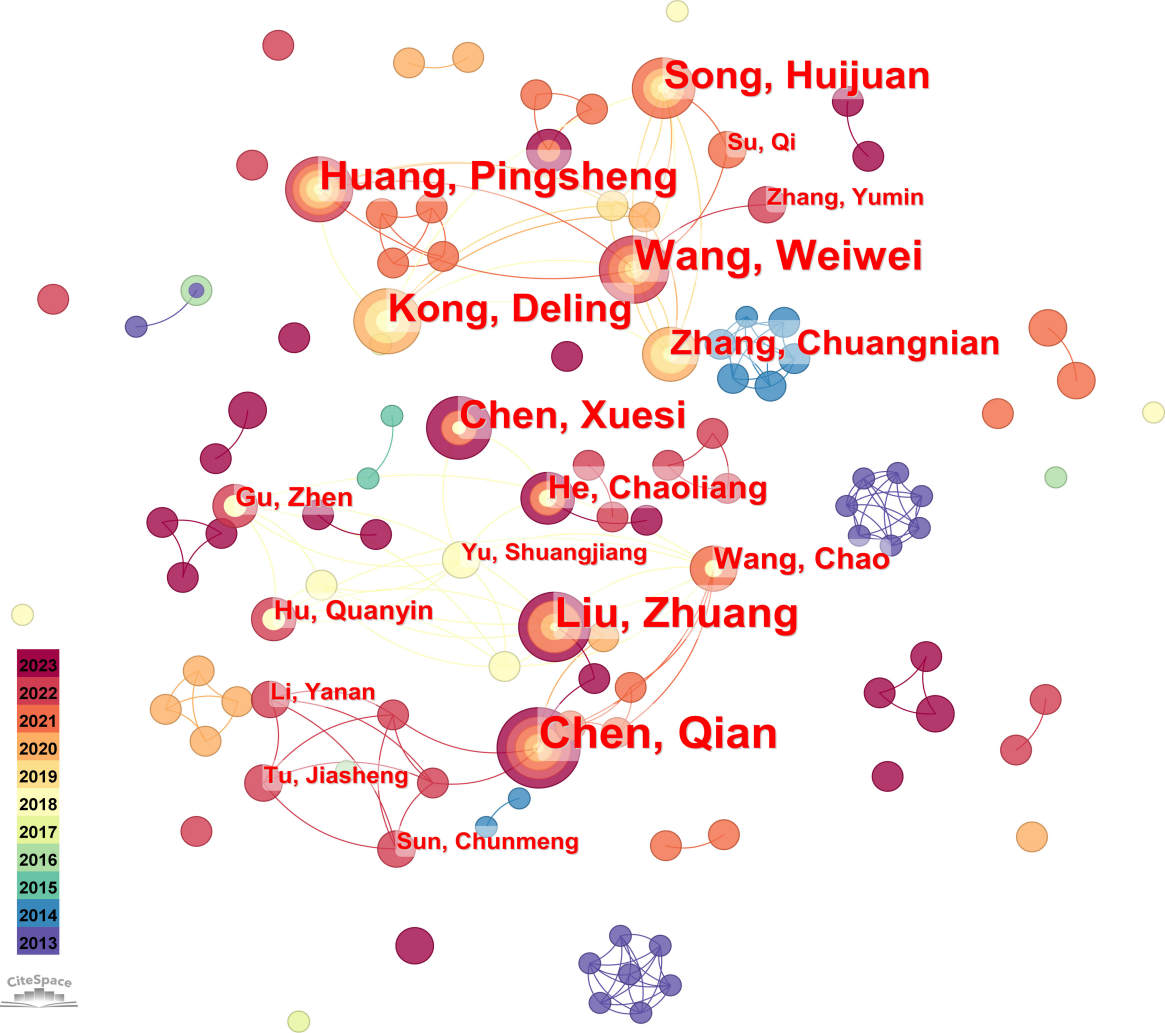
The co-occurrence map of authors.

In the present study, by selecting the author as the object of analysis in CiteSpace, with a time slice (years per slice) of 1 and a threshold of g-index = 10, we obtained an author analysis map with 152 network nodes, 206 connecting lines, and a density of 0.01. The graph of authors is similar to that of institutions, as presented in **Figure 4**. Both graphs show a concentration of nodes consisting of small groups of individual authors collaborating. Additionally, authors specialized in hydrogels cancer immunotherapy frequently collaborated. According to Price’s Law, the total number of authors engaged in hydrogels cancer immunotherapy research from 2013 to 2023 was 2,864, and its square root was 53.5, indicating that the number of core authors in hydrogels cancer immunotherapy was 54. Statistically, the number of publications by these core authors was 316, accounting for approximately 74.9% of the total publications. This is significantly greater than 50%, indicating that a stable core group of authors has established collaboration in hydrogels cancer immunotherapy.

**Figure 5:**
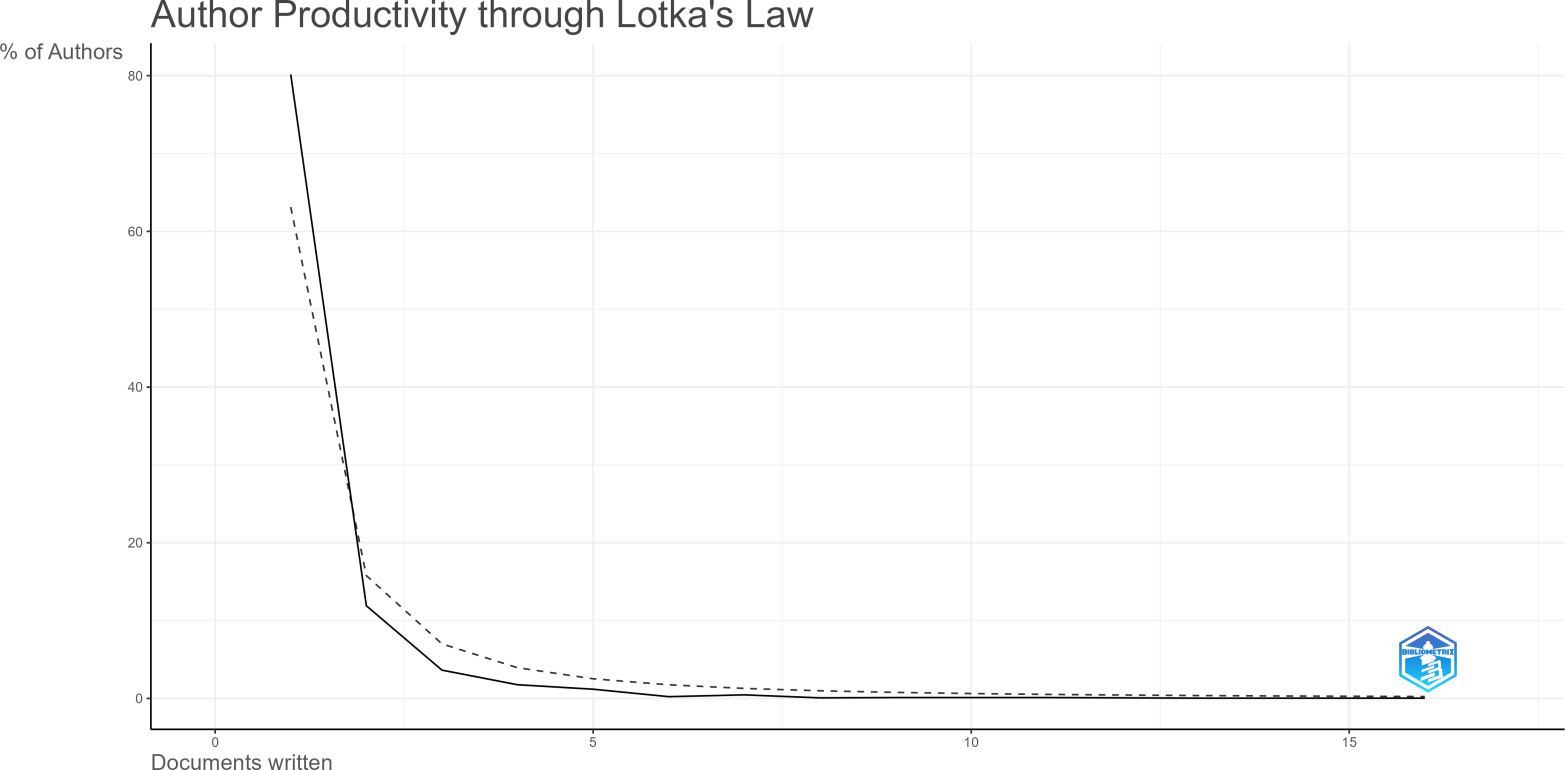
Author productivity using Lotkaw’s law. Solid black line indicates the distribution of published articles according to Lotkaw’s law. The dotted line indicates the publication on subject matter.

The researchers in hydrogels cancer immunotherapy have formed a collaborative team represented by Chen. Q, Liu. Z, Wang, W, Huang, P, and others, as demonstrated in **Figure 4**. Chen Qian’s group had its first publication in 2019, suggesting that despite commencing the research belatedly, it achieved a substantial quantity of findings throughout its duration, thereby making significant contributions to the field (**Table 3**). Liu. Z ranked second in terms of the number of publications, with a value of 12, and had some collaboration with Chen. Q. As evidenced by the prolific authors, the top ten authors in terms of the number of publications emerged after 2018, indicating that the research conducted by these prolific authors is the current cutting-edge research. Additionally, as shown by the centrality degree, Chen, Q., Liu, Z., and Wang, C have centrality degrees of 0.01 and 0.02, indicating that these three authors have a more extensive collaboration network in this field. Overall, Chinese scholars have the most research in this field, and there is an urgent need to increase the awareness of academic cooperation among scholars from other countries. It is necessary to establish a core team and increase academic influence in the field of hydrogels cancer immunotherapy. The productivity of authors was analyzed using Lotka’s law, as shown in **Figure 5**. The pattern of the number of publications follows Lotka’s law.

**Table 3:** The top 10 journals with the most publications.

Rank	Journals	Publications	Percentage	IF
1	Biomaterials	30	7.11%	14
2	Advanced Functional Materials	24	5.69%	19.2
3	Journal of Controlled Release	24	5.69%	10.8
4	Advanced Materials	19	4.50%	30.2
5	ACS Applied Materials Interfaces	16	3.79%	9.6
6	Biomaterials Science	15	3.55%	6.8
7	Acta Biomaterialia	11	2.61%	9.9
8	Advanced Healthcare Materials	10	2.37%	10.6
9	Advanced Science	10	2.37%	15.6
10	Chemical Engineering Journal	10	2.37%	15.1

#### 3.2.4 Analysis of academic journals

**Figure 6:**
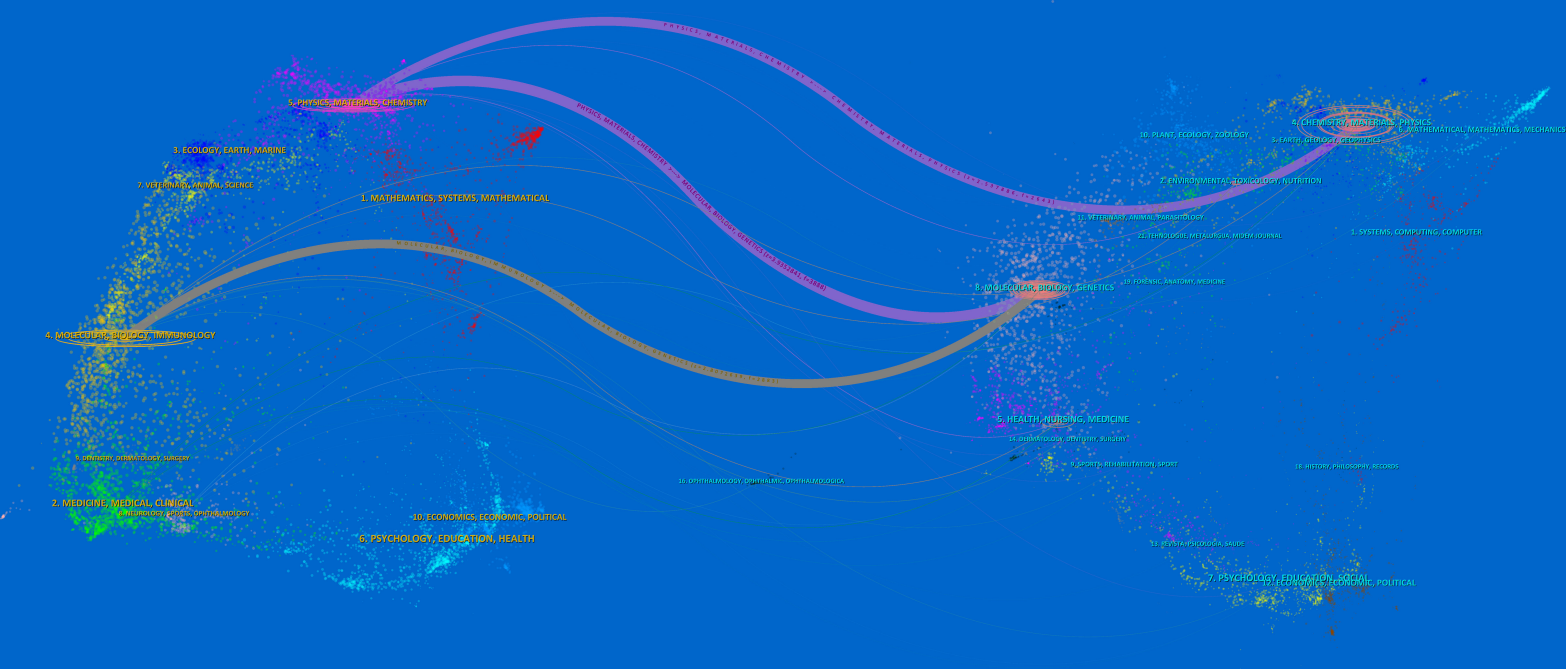
The dual-map overlay of journals on research of hydrogels in cancer immunotherapy.

**Figure 7:**
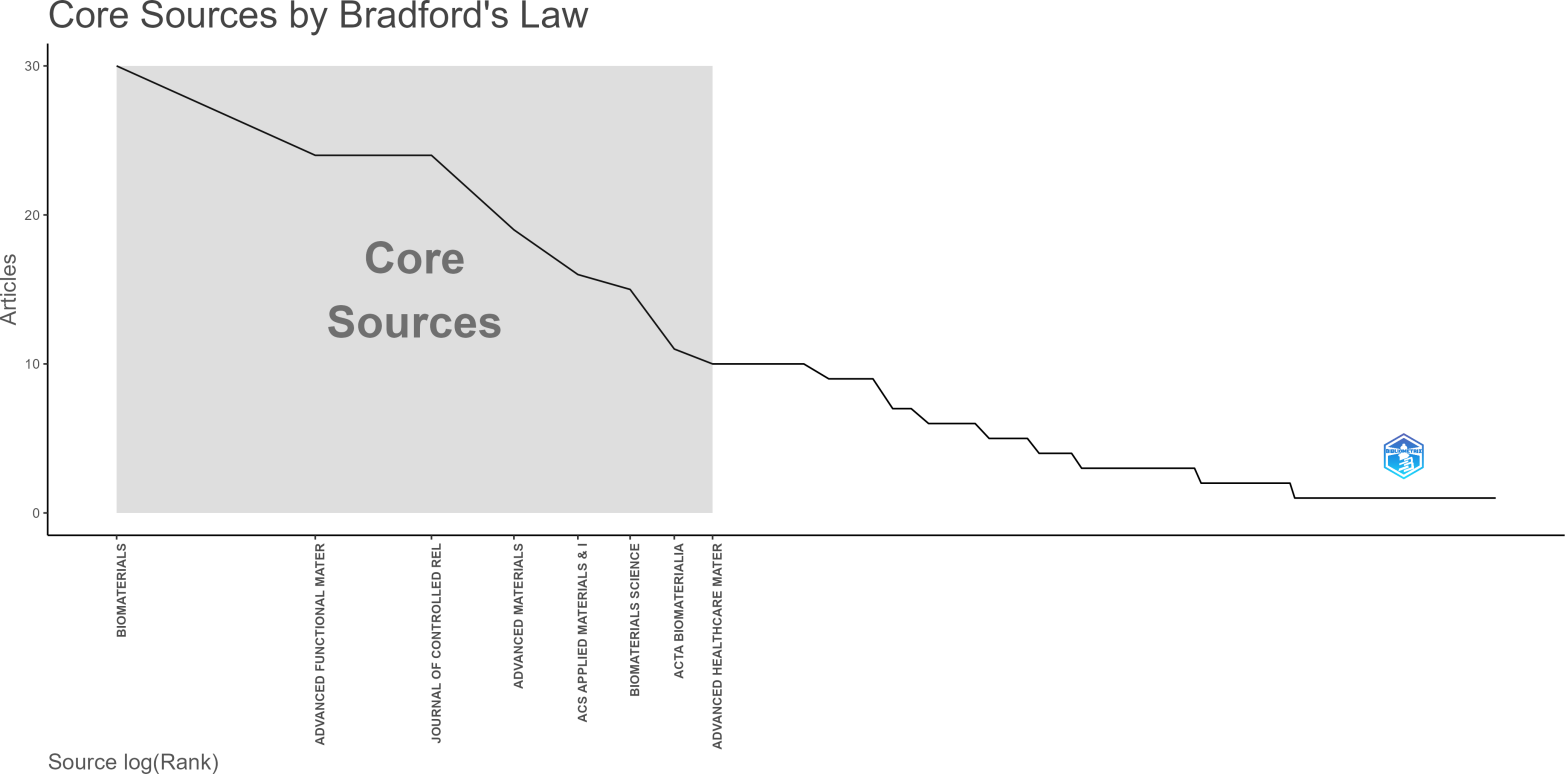
Bradford’s law applied to the set of publications.

The dual-image overlay function is to superimpose an atlas from CiteSpace software on top of another atlas; the former is known as an iterative atlas and the latter is known as an underlay atlas. The dual-map overlay function reflects the flow of knowledge between the cited literature and the administered literature. This study generated an overlay atlas of journals in hydrogels cancer immunotherapy based on the overlay function of CiteSpace, as illustrated in **Figure 6**. **Figure 6** is a dual-map overlay of journals, which can provide a more visual representation of the distribution of individual academic journals, the development of citation trajectories, and the change in research focus. The left half of **Figure 6** displays the distribution of the disciplines of the applied cited literature as the current research status of hydrogels cancer immunotherapy. The right half shows the disciplines of the cited literature as the research basis of hydrogels cancer immunotherapy. The wave curve connects the relationship between the current status of research and the research basis. The inner numbers of the ellipse indicate the number of publications in each discipline. The distribution of studies on the application of hydrogels in cancer immunotherapy research is primarily observed in journals that are cited in **Figure 6**. This provides insight into the knowledge flow within the WoS database concerning hydrogels cancer immunotherapy journals. Research in hydrogels cancer immunotherapy has focused primarily on molecular biology, immunology, mathematics, systematics, physics, materials science, and chemistry. The citations in hydrogels cancer immunotherapy are primarily concentrated in the journal clusters of molecular biology, genetics, chemistry, materials science, and physics. There are two outward citation paths in chemistry, materials science, and physics in the sizing domain on the left side of the figure, and this taxon is the most dominant sizing taxon. Furthermore, when the chemistry, materials science, and physics groups were used as source journals, the molecular science, biology, and genetics groups had the highest number of citations, with the highest Z value of 3.94. The Bradford’s laws applied for academic publications, which means that the most relevant publications are concentrated in a relatively small group of journals, as shown in **Figure 7**.

In this research, the distribution status of the top ten journals within hydrogels cancer immunotherapy research was determined by Excel (**Table 4**). The journals with more than 20 articles on hydrogels cancer immunotherapy research included Biomaterials (30 articles), Advanced Functional Materials (24 articles), and Journal of Controlled Release (24 articles). The percentage of publications in the top ten journals was 40.05%, which is less than 50%, indicating that the distribution of the literature on hydrogels cancer immunotherapy is relatively dispersed in various journals. Furthermore, the top ten journals were primarily involved in the fields of biology, materials, chemistry, and healthcare. These findings indicate that various scholars are deepening and enriching hydrogels cancer immunotherapy from different perspectives and directions, and research on this topic is more diverse and comprehensive. In terms of the impact factor, the average impact factor of the top ten journals in terms of the number of publications reached 14.18, which indicates that authoritative journals are paying more attention to research on hydrogels cancer immunotherapy.

**Table 4:** The top 20 keywords with the highest frequency.

Rank	Counts	Centrality	Year	Keywords
1	57	0.24	2014	cancer immunotherapy
2	24	0.2	2018	drug delivery
3	19	0.18	2021	immunogenic cell death
4	16	0.03	2021	tumor microenvironment
5	12	0.18	2016	injectable hydrogel
6	12	0.17	2019	immune checkpoint blockade
7	9	0.08	2022	photothermal therapy
8	8	0.04	2014	breast cancer
9	8	0.14	2019	photodynamic therapy
10	7	0.02	2022	tumor immunotherapy
11	6	0.05	2016	combination therapy
12	6	0	2013	dendritic cells
13	6	0.02	2021	controlled release
14	5	0.19	2020	cancer vaccine
15	5	0.11	2013	thermosensitive hydrogel
16	4	0.02	2022	NK cells
17	4	0.08	2021	sustained release
18	4	0.06	2022	solid tumor
19	3	0.02	2021	3d bioprinting
20	3	0	2017	hepatocellular carcinoma

### 3.3 Analysis of research hotspots

#### 3.3.1 Network analysis of keywords

**Figure 8:**
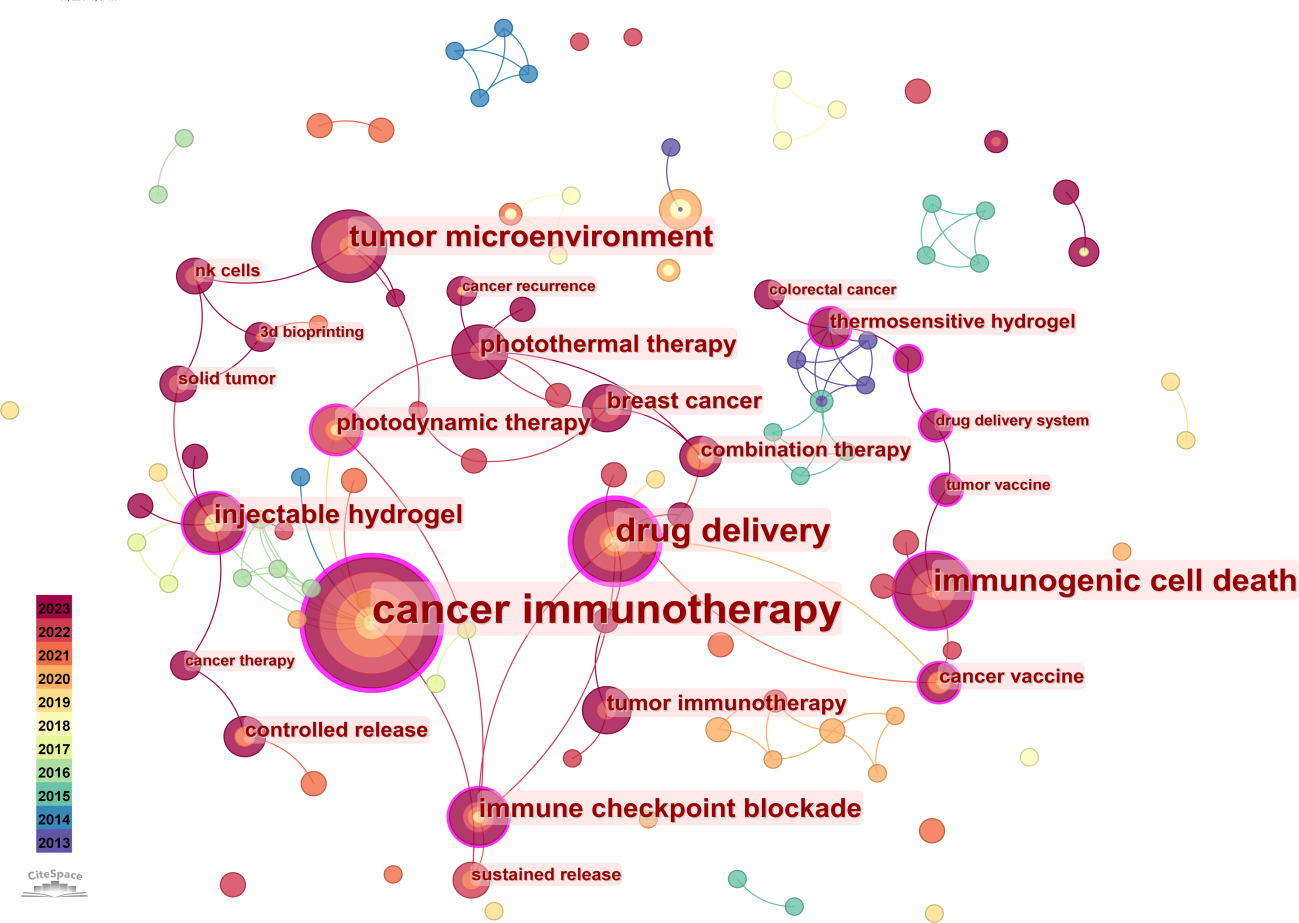
Visualization map of the keyword co-occurrence network.

In our study, CiteSpace software was used to analyze keywords in hydrogels in cancer immunotherapy. Keyword co-occurrence mapping was generated by selecting keywords with a time slice (years per slice) of 1, which eventually yielded 123 nodes, 121 contiguous lines, and a density of 0.0161 (**Figure 8**). The larger keyword nodes included cancer immunotherapy, drug delivery, immunogenic cell death, tumor microenvironment, injectable hydrogels, and immune checkpoint blockade. The six keywords, currently hot topics in hydrogels in cancer immunotherapy, primarily comprise measures and factors, including cancer immunotherapy, drug delivery, immunogenic cell death, the tumor microenvironment, injectable hydrogels, and immune checkpoint blockade. Furthermore, our study listed the top 20 keywords in terms of frequency (**Table 5**). **Table 5** demonstrates that cancer immunotherapy, drug delivery, immunogenic cell death, tumor microenvironment, injectable hydrogels, and immune checkpoint blockade are common, with frequencies of 57, 24, 19, 16, 12, and 12, respectively. The high-frequency themes focused on research topics, including immunotherapy, hydrogels materials, and immune cell adaptive environment. Cancer immunotherapy is in first place with a value of 0.24, which indicates a central position within hydrogels in cancer immunotherapy and is a core element of research in this field where cancer is the predominant disease. The keyword with the second highest centrality is drug delivery, with a value of 0.2, which indicates that scholars have also carried out a great deal of discussion around drug-controlled release. The keyword with the third highest centrality is a cancer vaccine, with a value of 0.19, suggesting that the use of hydrogels in cancer immunotherapy vaccines is also an important initiative to prevent cancer.

**Table 5:** Cluster information.

Cluster number	Number of nodes	Average profile value	Average year	Keywords content
0	14	0.91	2021	immunogenic cell death; tumor vaccine; immune check
1	12	1	2017	cancer immunotherapy; sustained release; photodynamic
2	10	0.986	2020	injectable hydrogel; cancer therapy; adhesive hydrogel
3	9	1	2014	bacillus calmette-guerin; chitosan; antitumor efficacy
4	8	0.893	2021	3d bioprinting; tumor microenvironment; NK cells
5	7	0.897	2019	photothermal therapy; breast cancer; combination therapy
6	6	1	2020	lymph nodes; adoptive cell therapy; cytokines;

#### 3.3.2 Cluster analysis of keywords

**Figure 9:**
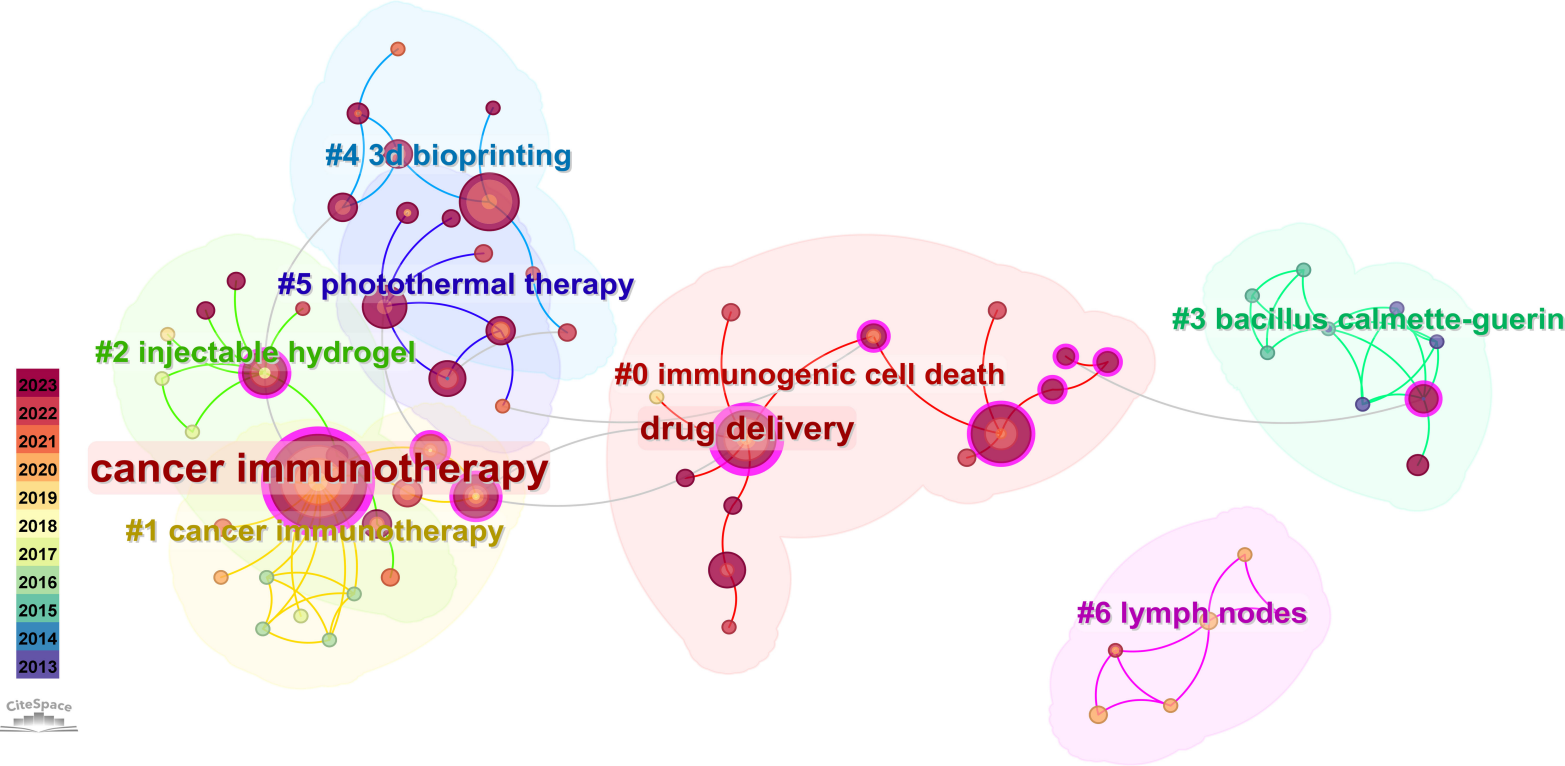
Cluster analysis of keywords.

In our study, cluster analysis of keywords was performed through CiteSpace, as shown in **Figure 9**, which indicates the research topics of hydrogels cancer immunotherapy in the last ten years. The cluster number is the topic of the keywords after clustering using the Log-Likelihood Ratio algorithm, and a total of nine clusters were obtained. The information on each cluster is shown in **Table 6**. The Q-value and the S-value are the metrics describing the structure of the network and clustering. This study generated results of Q = 0.8627 and S = 0.9614, which fulfills the criteria of the study. Therefore, the CiteSpace clustering results are reliable for subsequent analyses.

**Table 6:** Top 15 keywords with the strongest citation bursts (2013–2023).

Keywords	Strength	Begin	End	2013–2023
Bacillus calmette-guerin	1.29	2013	2015	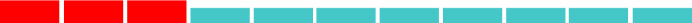
CpG motif	1.2	2017	2018	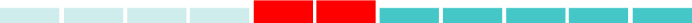
Dendritic cells	2.62	2018	2020	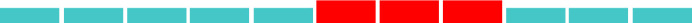
Cell encapsulation	1.41	2018	2020	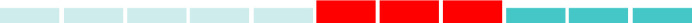
Cancer vaccines	1.12	2018	2019	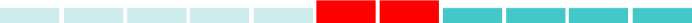
Adoptive cell transfer	0.94	2018	2020	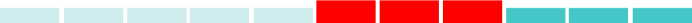
3D culture	0.61	2018	2021	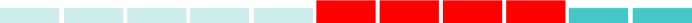
CpG nanoparticles	1.05	2019	2020	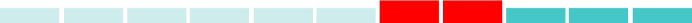
Adoptive T-cell therapy	0.72	2019	2021	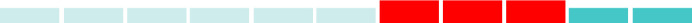
Black phosphorus	0.72	2019	2021	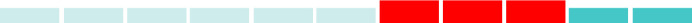
Cell capture	0.72	2019	2021	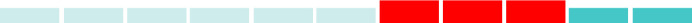
Adoptive cell therapy	0.66	2020	2023	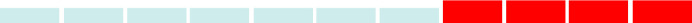
Tumor microenvironment	2.26	2021	2023	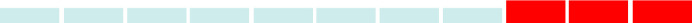
Photodynamic therapy	1.1	2021	2023	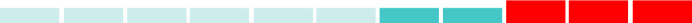
Sustained release	0.86	2021	2023	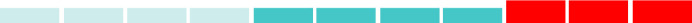

The information from seven clusters was counted in the study (**Table 6**). A total of seven research topics were generated in the study: #0 immunogenic cell death, #1 cancer immunotherapy, #2 injectable hydrogel, #3 bacillus calmette-guerin, #4 3D bioprinting, #5 photothermal therapy and #6 lymph nodes. As indicated by the content of the different keywords, the primary research content of the clusters included the effect of different hydrogels materials for cancer treatment in clinical trials, the adaptability of individual immune cell environments and the causes of cell death, and the therapeutic effect of different immunotherapeutic methods for cancer treatment. The average contour value of each cluster in the generated clusters is greater than 0.7, indicating that the impact of each cluster fulfills the requirements of the study. The average year suggests the average time of publication of the keywords within the clusters. The average year of 2014 for cluster 3 demonstrates that the research content of this cluster is more basic, suggesting that the earliest beginning of the study is hydrogels cancer immunotherapy based on the care of war-wounded individuals. Cluster 0 and Cluster 4 have an average year of 2021, indicating that the research topics of these two clusters are closer to the cutting edge.

### 3.4 Analysis of research evolution

#### 3.4.1 Research frontier

For this study, the burst detection algorithm of CiteSpace software was used to map keyword hotspot evolution, including keyword emergence, in hydrogels for cancer immunotherapy on the WoS (**Table 7**). The top 15 keywords related to the emergence intensity of hydrogels in cancer immunotherapy were identified in our study. The specific emergence intensities and durations of the hotspots are revealed in **Table 7**. The duration of research on hydrogels in cancer immunotherapy was 2013−2023. During the period of 2013−2016, the field was in the budding period, and scholars have focused on the hot topic of bacillus calmette-guerin, for which the intensity of emergence was 1.29. At the beginning of the study, scholars primarily investigated the use of the BCG vaccine in clinical trials to treat cancer disease through hydrogels. During 2016−2020, hydrogels cancer immunotherapy entered a stable period of development, and CpG motifs, dendritic cells, cell encapsulation, cancer vaccines, adoptive cell transfer, 3D culture, and CpG nanoparticles were the main research hotspots. The emergence intensities were 1.2, 2.62, 1.41, 1.12, 0.94, 0.61, and 1.05, respectively. The primary focus of research in this period was the adaptive environment under different types of cells. During the period of 2021−2023, adoptive T-cell therapy, black phosphorus, cell capture, adaptive cell therapy, tumor microenvironment, photodynamic therapy, and sustained release were the research hotspots in this field, and the emergence intensities were 0.72, 0.72, 0.72, 0.66, 2.26, 1.1 and 0.86, respectively. During this period, with the development of hydrogels application in cancer immunotherapy, many related research results were produced. This indicates that this field is relatively rich in research and is an important foundation stage for hydrogels cancer immunotherapy.

**Table 7:** The top 10 institutions with the most publications and greatest centrality.

Rank	Publications	Centrality	Year	Institution
1	52	0.17	2018	Chinese Academy of Sciences
2	32	0.01	2018	Chinese Academy of Medical Sciences
3	30	0.01	2018	Peking Union Medical College
4	23	0.24	2018	Soochow University - China
5	22	0.11	2018	Institute of BioMedical Engineering - CAMS
6	20	0.02	2019	Sichuan University
7	19	0.04	2022	Shanghai Jiao Tong University
8	16	0.06	2019	Fudan University
9	16	0.07	2020	University of Chinese Academy of Sciences
10	16	0.06	2018	Nankai University

#### 3.4.2 Trends in research

**Figure 10:**
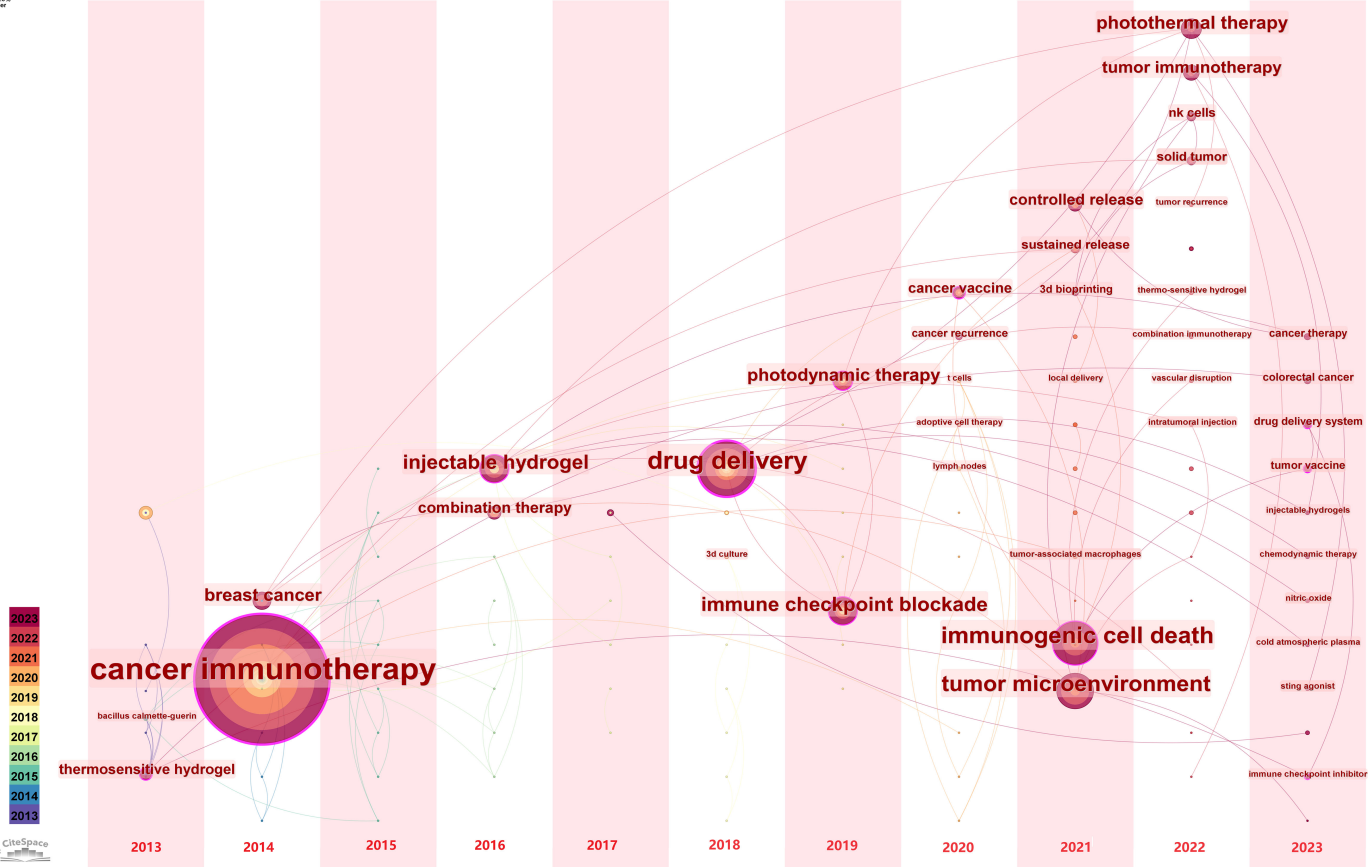
Time zone view of keywords.

By this study, we generated a time-series evolution graph for keywords of hydrogels in cancer immunotherapy on the WoS by selecting the time zone as the analysis node through CiteSpace using the keywords over time. The specific metrics and thresholds set were: the time slice of 1, the threshold is selected to be g-index = 10, and the keywords with smaller nodes are hidden, generating the current graph, and finally obtaining 123 nodes, 121 connecting lines, and a density of 0.0161, as depicted in **Figure 10**. The timeline describes the appearance of keywords over time, including the position corresponding to the keyword and the year in which the keyword first appeared. During the period 2013−2016, dendritic cells, thermosensitive hydrogels, cancer immunotherapy, breast cancer, and injectable hydrogels appeared in hydrogels for cancer immunotherapy research, combination therapy, and other research (**Figure 10**). This suggests that in the early stage of research, scholars in hydrogels for cancer immunotherapy primarily investigated dendritic cells, thermosensitive hydrogels, cancer immunotherapy, and other issues. During the period of 2017−2020, hydrogels cancer immunotherapy research appeared in hepatocellular carcinoma, drug delivery, immune checkpoint blockade, photodynamic therapy, cancer vaccine, cancer recurrence, T cells, and other research themes. During this period, scholars began to enhance the efficiency and effectiveness of hydrogels cancer immunotherapy by means of liver cancer, drug delivery, immune checkpoint blockade, and photodynamic therapy. During the period of 2021−2023, studies on hydrogels in cancer immunotherapy research appeared on immunogenic cell death, tumor microenvironment, controlled release, sustained release, 3D bioprinting, local administration, local delivery, photothermal therapy, cancer immunotherapy, NK cells, and solid tumor. Recently, this era of research has gained considerable interest in hydrogels cancer immunotherapy, including immunogenicity cell death, tumor microenvironment, controlled release, slow-release, 3D bioprinting, and other topics.

## 4 Discussion

### 4.1 General information

Cancer treatment is thorough and includes surgical procedures, chemotherapy, radiation therapy, targeted therapy, and immunotherapy (23, 24). Recently, hydrogels have been displayed to play a significant role in cancer immunotherapy (25). Hydrogels have gained considerable interest due to their biocompatibility, environmental friendliness, and ease of production in the cancer immunology field (26).

In this paper, we utilized bibliometric technology to conduct a comprehensive analysis of studies related to hydrogels in cancer immunotherapy in terms of the published literature, authors, countries, journals, institutions, keywords, and research hotspots. A total of 422 publications on hydrogels in cancer immunotherapy research were included in the analysis from 2013 to 2023 by searching the WoS Core Collection. Research on the use of hydrogels in cancer immunotherapy showed a steady upward trend from 2013. A total of 35 countries, 146 institutions, and 152 authors contributed to this field. The two points, 2020−2021 and 2021−2022, were the moments of publication bursts, which indicated that more attention had been paid to this field.

At the global level, China and the United States have been the most productive in this field. **Table 1** shows that China has published the maximum number of research papers on hydrogels for cancer immunotherapy. Geographical analysis (**Figure 3**; **Table 2**) indicated China’s dominant role in related research concerning hydrogels for cancer immunotherapy. The three institutions with the most publications were the Chinese Academy of Sciences (52 publications), the Chinese Academy of Medical Sciences (32 publications), and Peking Union Medical College, Peking Union Medical College (30 publications). For the 146 institutions, the cooperation networks revealed that domestic institutions collaborated frequently, and they had obvious regional characteristics. Based on the number of publications, the top three journals were Biomaterials (30 publications), Advanced Functional Materials (24 publications), and Controlled Release (24 publications).

### 4.2 Hotspots and frontiers

Rapid and efficient determination of the distribution of hot spots can be achieved by analyzing the composition, distribution, and clustering of keywords. Cancer immunotherapy, drug delivery, immunogenic cell death, tumor microenvironment, injectable hydrogels, and immune checkpoint blockade represent the main research directions of hydrogels in cancer immunotherapy (27).

Cancer immunotherapy drugs have revolutionized the treatment of certain cancers by eliminating the body’s immune system to attack tumors, including lymphoma, lung cancer, and melanoma (28, 29). Conventional tumor treatments include surgery, radiation therapy, and chemotherapy. Cancer immunotherapy, a precisely targeted or individualized therapy, may be safer than traditional therapies (30, 31). On May 10, 2017, the U.S. Food and Drug Administration (FDA) expedited the approval of the PD-1 monoclonal antibody pembrolizumab in combination with pemetrexed and carboplatin for the first-line treatment of non-squamous cell lung cancer (NSCLC) (32, 33). This is the first approved indication for an immune checkpoint inhibitor in combination with chemotherapy and the third indication for Pembrolizumab for treating advanced NSCLC. In September 2017, the FDA approved Nivolumab for treating hepatocellular carcinoma after treatment with sorafenib (34). Nivolumab is the first and only FDA-approved immunotherapeutic for this purpose (35).

Effective targeted drug delivery is currently a bottleneck in treating diseases with drugs. In drug-delivery hydrogels, biodegradation can play a role in drug release (36). The drug delivery system (DDS) refers to the method of transporting drugs to the required tissues, organs, cells, and subcellular organs through various drug carriers for effective drug release and absorption (37). A drug delivery control system is essential for ensuring sustained and effective delivery of drugs with minimal side effects (38). The concept of immunogenic cell death (ICD) was introduced in 2005 to describe cell death that can elicit an immune response (39). Immunogenic cell death is a specific variant of regulatory cell death that is driven by stress and can induce adaptive immunity against dead cell antigens (40). A recent study demonstrated that immunogenic cell death can potentiate the immune checkpoint inhibitor treatment effect (41). The tumor microenvironment is a complex ecosystem containing different types of tumor cells, stromal cells, and immune cells (42–44). Interactions between these cell types can remodel the tumor microenvironment and regulate tumor progression (45). There is an important role for the tumor microenvironment in the development, progression, metastatic spread, and drug resistance of cancers (46). It has emerged that immunotherapy is a highly effective therapy for cancer by harnessing the immune cells found in tumor microenvironments (47).

Injectable hydrogels are a new type of hydrogel (48). Injectable hydrogels are water-soluble polymer solutions that undergo *in situ* gelation when applied to a target environment, such as the human body or a porous scaffold (49). It is a pre-injection liquid that is injected with a standard syringe during treatment and can be injected after injection. The hydrogels can be injected into almost any desired location, allowing the drug to accumulate in the local site and effectively reducing systemic toxicity (50). Hydrogels can be loaded with drugs from genes, cytokines, peptide vaccines, antibodies, and even cells, which is in line with the current development trend of cancer immunotherapy and has a wide range of application values. Xiao. Z et al. developed a PEIGel hydrogel that can be used for local injection. The gel has an immune-assisted function and can synergistically interact with the immune drugs loaded in the gel to promote the effect of cancer immunotherapy, making it suitable for more patients (51). Wen. J et al. developed a new type of injectable hydrogel for the unique tumor microenvironment of pancreatic cancer (52). Their study on animal models proved that the new hydrogels could ablate tumor cells through rapid local photothermal therapy, regulate the body’s immunity, and effectively inhibit the progression and metastasis of pancreatic cancer. Feng. Q et al. developed an injectable sericin (SS)/silk fibroin (SF) recombinant hydrogel, called SF-SS-SMC hydrogel, to achieve local delivery of an anti-CD47 antibody (αCD47) (53). Based on the combined effect of the sustained release of αCD47 and TME reprogramming, SF-SS-SMC hydrogel has sound immunotherapy effects on local, distant, remitted, and metastatic tumors. Furthermore, its ease of use, low cost of production, and straightforward manufacturing process all contribute to its anticipated application in commercial batch production.

Regulatory molecules such as immune checkpoints inhibit the immune system and are involved in immune regulation (54). Under normal circumstances, immune checkpoints can maintain immune tolerance by regulating the intensity of autoimmune responses (55, 56). However, when tumors invade the body, tumor cells inhibit the body’s immune surveillance and clearance of tumor cells by abnormally activating immune checkpoints to achieve tumor cell proliferation and escape. There is currently a wide range of immunotherapy options available, including checkpoint blockade and chimeric antigen receptor T-cell therapy (57). In the last decade, immune checkpoint blockade has emerged as a powerful tool for controlling pathogenic immune responses (58). At present, the primary drugs used for immune checkpoint blockade are PD-1/PD-L1, and many pharmaceutical companies in China have announced relevant clinical trials (59, 60). In addition, the hydrogels can significantly activate cellular and humoral immune responses (61). As cancer immunotherapy progresses, immune checkpoints become increasingly important (62).

Cluster analysis of the keywords revealed the following main categories: immunogenic cell death, cancer immunotherapy, injectable hydrogel, bacillus calmette-guerin, 3D bioprinting, photothermal therapy, and lymph nodes. Yang. T et al. innovatively developed a crosslinked nanoparticle composite hydrogel for preventing surgical bleeding and recurrence of hepatocellular carcinoma (63). They finally formed an injectable fibrin hydrogel through the reaction of fibrinogen and thrombin. They dispersed the antibody-loaded nanoparticles to prepare a nanocomposite hydrogel with hemostatic function and slow-release drug properties. Zhu. YQ et al. developed a βCD decorative alginate (ALG-βCD) hydrogel with T-cell recruitment, engagement, and regeneration to enhance T-cell-mediated immunotherapy (64). Photodynamic therapy is a localized treatment for tumors that kills tumor cells by generating reactive oxygen species (ROS) from photosensitizers under light exposure (65). This addresses the obstacle that limits the clinical efficacy of immunotherapy with T cells (TCBI). Zhang et al. designed for the first time a chemically modified tumor lysate-constructed hydrogels that created a powerful immune ecological niche within the tumor and achieved efficient inhibition of tumor growth, metastasis, and recurrence (66).

Bacillus Calmette guerin vaccine: obtained from Mycobacterium bovis isolates; used for tuberculosis vaccination. The bacillus calmette Guerin vaccine, which was initially used to prevent disease, elicits an immune response when injected into the bladder (67). In additional, bacillus calmette guerin has been used for bladder cancer immunotherapy for nearly half a century (68). In traditional antitumor therapies, photothermal therapy is one of the most common methods. Photothermal therapy involves irradiating tumor cells with light at a specific wavelength, which heats them up (69). Photothermal therapy is known to eliminate primary tumors and trigger a systemic antitumor immunity (70). Light radiation generates local heat in cancer cells during photothermal therapy (71). Immune responses may be enhanced by photothermal therapy in synergy with immunotherapy. Lymph nodes are an important part of cancer immunotherapy because lymph node-derived immune cells play a role (72). Lymph nodes return to a steady state after being exposed to an immune response (73).

As evidenced by the analysis of research hotspots, the focus of hydrogels in cancer immunotherapy research has evolved. The bio3D printing technology also offers unique advantages in mimicking natural tissue structures by building layers of cell-containing bioink on a predetermined path to form 3D tissues and organs with complex structures (74). Zhong. RB et al. showed that the hydrogel system is capable of sustained localized delivery of RNA (avoiding repeated dosing) and spatial and temporal control of the release rate. Hydrogel microspheres are a class of hydrogels with micrometer sizes that have a wide range of applications in the biomedical field. Recently, there has been a gradual increase in research on the application of hydrogel microspheres as bio-inks for bio3D printing (75, 76).Traditionally, targeting technology has been limited due to its low efficiency. As carriers, hydrogels are highly efficient and capable of transporting anticancer drugs, photosensitizers, and immune stimulants (77, 78). Mechanical tunability, rapid gelation, biocompatibility, and biodegradability were all demonstrated by these hydrogels (79, 80). In spite of their many benefits, hydrogels have also some limitations and risks associated with their use in drug delivery systems. The application of hydrogels in cancer immunotherapy are in its infancy. Drug loading and delivery capability are directly influenced by hydrogels’ ability to absorb water (swelling behavior) (81). There is also the issue of some hydrogels releasing drugs rapidly and early (82). It is necessary to control drug release rate accurately by modifying hydrogel properties. Currently, there are not many studies on this topic. This approach may provide a novel strategy for cancer immunotherapy.

### 4.3 Limitations

There are some limitations in our study. Firstly, all the data rely on papers from the WoS. Even though the WoS database contains authoritative and influential journals, some publications from other database, may not be included in this study. Secondly, the search available literature was carried out only in the current decade. However, our analysis focused on the latest knowledge gaps and areas for further investigation. Finally, only publications written in English were included. Despite these limitations, the bibliometric analysis provides much better insight into the research trends and hotspots of research on hydrogels in cancer immunotherapy.

## 5 Conclusion

Hydrogels are anticipated to be promising antitumor drug delivery systems as research on hydrogels anticancer immunotherapy continues to progress. With the in-depth study of hydrogels and the continuous improvement of immunotherapy and other combined treatments, hydrogels will become potential drug carriers. The properties of hydrogels can be utilized to ameliorate mild and transient immune responses in the host. Additionally, hydrogels can be engineered to exhibit diverse environmental sensitivities, and their immunogenicity can be improved by integrating the mechanisms of chemotherapeutic drug-induced immunogenic cell death. Therefore, hydrogel systems can be used as slow-release systems for chemotherapy and as carriers for cancer vaccines. Moreover, they reduce the toxic side effects of conventional chemotherapy and enhance the antitumor effects of tumor vaccines. This research has the potential to improve our comprehension of hydrogels anticancer immunotherapy and serve as a source of inspiration for subsequent investigations.

## Data Availability

The raw data supporting the conclusions of this article will be made available by the authors, without undue reservation.
